# Determinants of Human African Trypanosomiasis Elimination via Paratransgenesis

**DOI:** 10.1371/journal.pntd.0004465

**Published:** 2016-03-08

**Authors:** Jennifer A. Gilbert, Jan Medlock, Jeffrey P. Townsend, Serap Aksoy, Martial Ndeffo Mbah, Alison P. Galvani

**Affiliations:** 1 Center for Infectious Disease Modeling and Analysis, Yale School of Public Health, New Haven, Connecticut, United States of America; 2 Epidemiology of Microbial Diseases, Yale School of Public Health, New Haven, Connecticut, United States of America; 3 Department of Biomedical Sciences, Oregon State University, Corvallis, Oregon, United States of America; 4 Department of Biostatistics, Yale University, New Haven, Connecticut, United States of America; 5 Department of Ecology and Evolutionary Biology, Yale University, New Haven, Connecticut, United States of America; 6 Program in Computational Biology and Bioinformatics, Yale University, New Haven, Connecticut, United States of America; University of California Berkeley, UNITED STATES

## Abstract

Human African trypanosomiasis (HAT), transmitted by tsetse flies, has historically infected hundreds of thousands of individuals annually in sub-Saharan Africa. Over the last decade, concerted control efforts have reduced reported cases to below 10,000 annually, bringing complete elimination within reach. A potential technology to eliminate HAT involves rendering the flies resistant to trypanosome infection. This approach can be achieved through the introduction of transgenic *Sodalis* symbiotic bacteria that have been modified to produce a trypanocide, and propagated via *Wolbachia* symbionts, which confer a reproductive advantage to the paratransgenic tsetse. However, the population dynamics of these symbionts within tsetse flies have not yet been evaluated. Specifically, the key factors that determine the effectiveness of paratransgenesis have yet to be quantified. To identify the impact of these determinants on *T*.*b*. *gambiense* and *T*.*b*. *rhodesiense* transmission, we developed a mathematical model of trypanosome transmission that incorporates tsetse and symbiont population dynamics. We found that fecundity and mortality penalties associated with *Wolbachia* or recombinant *Sodalis* colonization, probabilities of vertical transmission, and tsetse migration rates are fundamental to the feasibility of HAT elimination. For example, we determined that HAT elimination could be sustained over 25 years when *Wolbachia* colonization minimally impacted fecundity or mortality, and when the probability of recombinant *Sodalis* vertical transmission exceeded 99.9%. We also found that for a narrow range of recombinant *Sodalis* vertical transmission probability (99.9–90.6% for *T*.*b*. *gambiense* and 99.9–85.8% for *T*.*b*. *rhodesiense*), cumulative HAT incidence was reduced between 30% and 1% for *T*.*b*. *gambiense* and between 21% and 3% for *T*.*b*. *rhodesiense*, although elimination was not predicted. Our findings indicate that fitness and mortality penalties associated with paratransgenic symbionts, as well as tsetse migration rates, are instrumental to HAT elimination, and should be a key focus in the development of paratransgenic symbionts.

## Author Summary

Human African trypanosomiasis, also known as sleeping sickness, is a parasitic disease transmitted by tsetse flies in sub-Saharan Africa. The disease leads to death if not treated. Recent control efforts have reduced the burden of disease from hundreds of thousands of cases per year to fewer than 10,000 cases annually. A potential strategy to completely eliminate sleeping sickness involves genetically modifying the symbiotic bacteria, which are vertically transmitted from mother to offspring, in order to investigate which factors are most important for the successful elimination of human African trypanosomiasis. We found that sleeping sickness was eliminated only when the genetically modified symbionts were successfully transmitted from mother to offspring, and did not reduce fertility or increase mortality in tsetse. We additionally identified tsetse migration rate as an important factor for sleeping sickness elimination.

## Introduction

Human African trypanosomiasis (*i*.*e*. HAT or sleeping sickness) has historically infected more than 300,000 individuals annually in sub-Saharan Africa [1]. Over the last decade, concerted control efforts have reduced the annual number of reported HAT cases to between 7,000 and 10,000 [2–4]. The vast majority of these cases result from infection with *Trypanosoma brucei gambiense*, which is found in West and Central Africa and causes a chronic form of the disease. The remainder of sleeping sickness cases arise from *Trypansoma brucei rhodesiense* infection, which is prevalent in East and South Africa and causes a more acute form of the disease. The WHO aims to eliminate HAT as a public health problem by 2020 [3].

Past and current HAT control efforts have focused on active surveillance, treatment of humans and animal reservoirs, and tsetse vector control. Patient-targeted strategies face many obstacles; there is no vaccine and treatment requires expensive and toxic intravenous drugs [5, 6]. Vector control strategies—including insecticide, traps, land clearing, and sterile male releases—have had some success in reducing the incidence of disease by controlling tsetse populations [5, 6]. However, to control or eliminate the disease, sustained strategies are required [5, 6]. Without such efforts, the disease reemerges, as evidenced by the tsetse and HAT resurgence that occurred in the 1980s following a lapse in control efforts [7]. A potential technology to control HAT involves rendering tsetse flies refractory to trypanosome infection via the introduction of genetically modified symbiotic bacteria [8–10]. In this paratransgenic technique, one of the symbionts, of the genus *Sodalis*, is modified to produce a trypanocidal molecule [11], while the other, of the genus *Wolbachia*, confers a reproductive advantage to the paratransgenic flies via cytoplasmic incompatibility [12]. It is *Wolbachia* that drives *Sodalis* into the tsetse population. This paratransgenic approach has the potential to be sustainable, because the recombinant symbionts are vertically propagated throughout the tsetse population from mother to offspring [8–10, 13].

Although this approach has been used to reduce the vectorial capacity of other insects including triatomine bugs (vector of *Trypanosoma cruzi)*, sandflies (vector of *Leishmania*), and mosquitoes (vector of malaria), paratransgenesis has not yet been implemented in the field as an intervention for tsetse [14–19]. Both the genetic modification of *Sodalis* and its vertical transmission from mother to offspring have been investigated in the laboratory, as has the ability of *Wolbachia* to generate cytoplasmic incompatibility within tsetse [12]. However, several questions remain around the dynamics of recombinant *Sodalis* and *Wolbachia* within the tsetse population as the intervention continues to be developed. In particular, it is not yet known if colonization with the new symbionts would impose any fitness penalties for tsetse fecundity or mortality, or how these penalties might affect HAT elimination. Furthermore, it is also not known how the migration of tsetse in and out of a targeted area—which is not yet quantified for many locations within sub-Saharan Africa—might dilute the impact of the intervention.

In a previous study, we developed a mathematical model that incorporated the population genetics of cytoplasmic incompatibility-inducing *Wolbachia* with the transmission dynamics of *T*.*b*. *gambiense* [13]. We used this model to demonstrate the potential for *Wolbachia-*colonized tsetse to establish themselves in a population of wild type tsetse. We also used the model to examine the reduction in *T*.*b*. *gambiense* HAT that would be observed if these *Wolbachia-*colonized tsetse were resistant to trypanosome infection. In the current study, we expanded our mathematical model of trypanosome transmission and tsetse population dynamics to incorporate the vertical transmission of both *Wolbachia* and *Sodalis* (wild type and recombinant) symbionts, as well as transmission of both *T*.*b*. *gambiense* and *T*.*b*. *rhodesiense*. We parameterized our model using existing data from the epidemiological and entomological literature for *T*.*b*. *gambiense* and *T*.*b*. *rhodesiense*, respectively. To evaluate the contributors to HAT elimination via paratransgenesis, we assessed the prevalences of *Wolbachia* and recombinant *Sodalis* reached within the tsetse population, the changes in the reproductive number of HAT, as well as the percentage reduction in HAT cases predicted over 25 years for varying *Wolbachia* and recombinant *Sodalis*-associated fecundity and mortality penalties, vertical transmission probabilities, and tsetse migration rates.

## Methods

### Model Description

**Fig 1:**
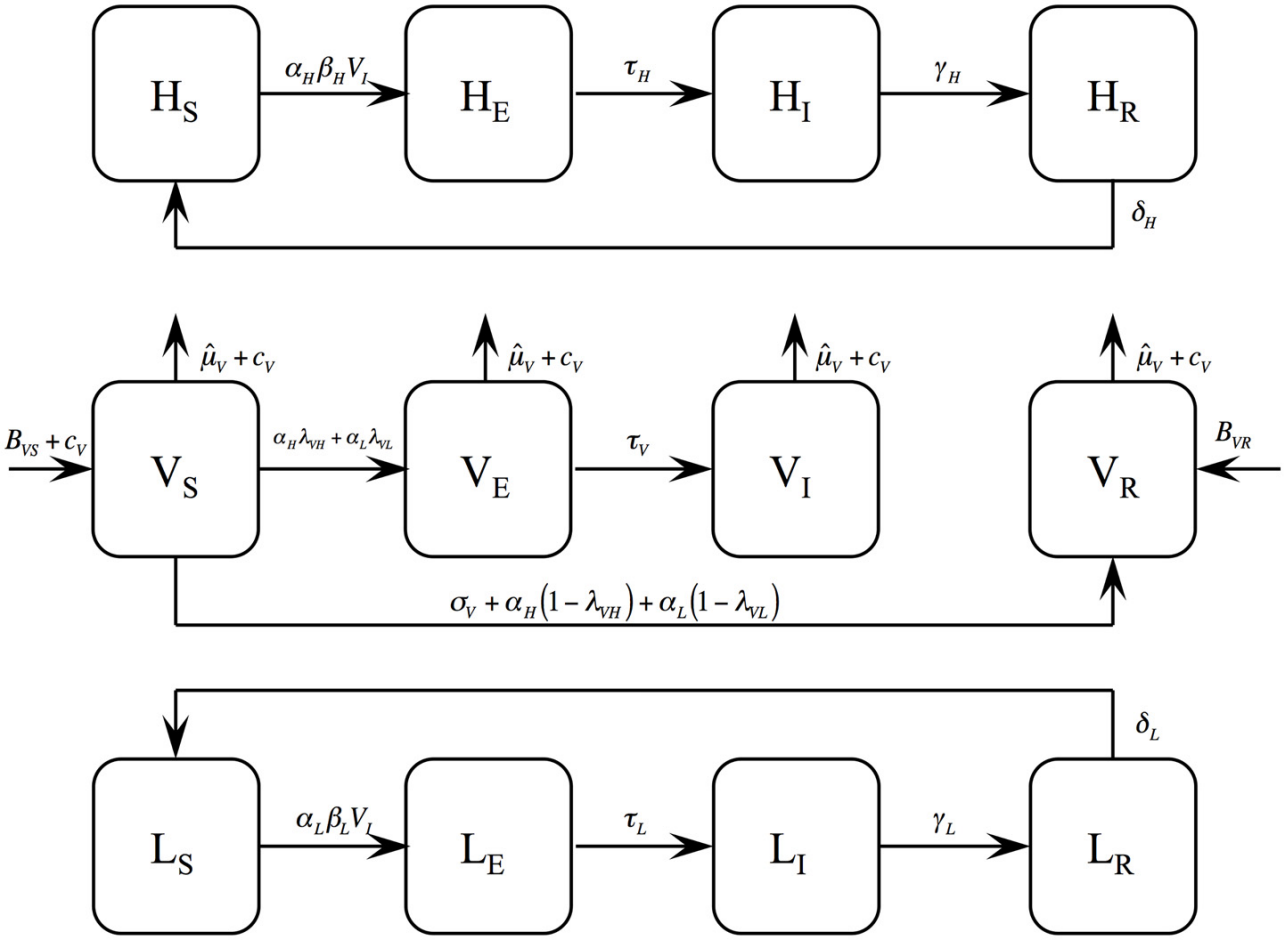
Model diagram. Compartmental model of African trypanosomiasis transmission between hosts (human [H] or livestock [L]) and tsetse fly [V] vectors. Hosts are susceptible [S], latently infected [E], infectious [I] or recovered [R]. Vectors are susceptible [S], latently infected [E], infectious [I] or resistant [R].

To project the temporal dynamics of the paratransgenesis symbionts and the proportion of HAT cases over 25 years, we extended our previous model of trypanosome transmission [13] to include tsetse and symbiont population dynamics (Fig 1, see S1 Text for model equations). The model was parameterized to reflect *T*.*b*. *gambiense* or *T*.*b*. *rhodesiense* transmission (see Table 1 for parameter values), respectively, with two major distinctions between the trypanosome species: the infectious period of *T*.*b*. *gambiense* is much longer than that of *T*.*b*. *rhodesiense*, and the tsetse vectors of *T*.*b gambiense* have a greater preference for feeding on humans versus animals than the vector of *T*.*b*. *rhodesiense*. Thus, in the absence of any intervention, the prevalence of *T*.*b gambiense* within humans relative to that of *T*.*b*. *rhodesiense* is higher in the model, consistent with observations in sub-Saharan Africa [2].

**Table 1:** Model parameters of both *Trypanosoma brucei* subspecies.

	Parameter	*T*.*b*. *gambiense*		Source	*T*.*b*. *rhodesiense*		Source
		Value	Range		Value	Range	
*r*_V0_	Tsetse fecundity (per day)	0.02	0.01–0.03	[28]	0.02	0.01–0.03	[28]
*r*_V1_	Fecundity density-dependence parameter^a^	0	0–0.0005	–	0	0–0.0005	–
*r*_W_	Fecundity penalty for *Wolbachia*	0	–0.5–0.5	–	0	–0.5–0.5	–
*r*_Sr_	Fecundity penalty for recombinant *Sodalis*	0	0–0.05	–	0	0–0.05	–
*r*_Sn_	Fecundity penalty for no *Sodalis*	0	0–0.5	–	0	0–0.5	–
*μ*_V0_	Tsetse mortality rate (per day)	0.01	0.005–0.015	[28]	0.01	0.005–0.015	[28]
*μ*_V1_	Mortality density-dependence parameter^a^	0.0002	1x10^−5^–5x10^-4^	–	0.0002	1x10^−5^–5x10^-4^	–
*μ*_W_	Mortality rate increase due to *Wolbachia*	0	–0.1–0.1	–	0	–0.1–0.1	–
*μ*_Sr_	Mortality rate increase due to recombinant *Sodalis*	0	0–0.1	–	0	0–0.1	–
*μ*_Sn_	Mortality rate increase due to no *Sodalis*	0	0–0.1	–	0	0–0.1	–
*ϕ*_W_	Maternal transmission failure probability for *Wolbachia*	0.05	0–0.2	[12]	0.05	0–0.2	[12]
*ϕ*_Sr_	Maternal transmission failure probability for recombinant *Sodalis*	0	0–0.1	–	0	0–0.1	–
*ϕ*_Sw_	Maternal transmission failure probability for wild type *Sodalis*	0	0–0.01	–	0	0–0.01	–
*h*_W_	Cytoplasmic incompatibility hatch failure probability	1	0.7–1	[12]	1	0.7–1	[12]
*H*	Human population size	300	100–1000	[28–34]	300	100–1000	[28–34]
*L*	Animal population size	50	10–500	[28, 34–37]	50	10–500	[28, 34–37]
V*	Equilibrium tsetse population size	5000	–	[28]	5000	–	[28]
*ψ*_Vw_	Fraction of wild type-*Sodalis*-positive tsetse that are inherently immune	0	0–1	–	0	0–1	–
*ψ*_Vn_	Fraction of *Sodalis*-negative tsetse that are inherently immune	0	0–1	–	0	0–1	–
*a*_H_	Tsetse human biting rate (per day)	0.1	0.013–0.143	[28–31, 34–44]	0.0167	0.0025–0.06	[28–31, 34–44]
*a*_L_	Tsetse animal biting rate (per day)	0.233	0.093–0.317	[28–30, 33–39]	0.317	0.205–0.33	[28–30, 33–39]
*β*_VH_	Transmission probability from humans to tsetse	0.065	0.05–0.14	[28–30]	0.065	0.05–0.14	[28–30]
*β*_VL_	Transmission probability from animal to tsetse	0.065	0.033–0.22	[28, 36]	0.065	0.033–0.22	[28, 36]
*β*_H_	Transmission probability from tsetse to humans	0.62	0.1–0.62	[28–30, 38]	0.62	0.1–0.62	[28–30, 38]
*β*_L_	Transmission probability from tsetse to animal	0.62	0.22–0.61	[28, 32, 33, 38]	0.62	0.22–0.61	[28, 32, 33, 38]
*σ*_V_	Susceptibility period in tsetse (days)	1	0.5–30	[28]	1	0.5–30	[28]
*τ*_V_	Incubation time in tsetse (days)	25	15–30	[28–30, 34]	25	15–30	[28–30, 34]
*τ*_H_	Incubation time in humans (days)	12	5–15	[28–30]	12	5–15	[28–30]
*τ*_L_	Incubation time in animal (days)	12	5–15	[28, 32, 33]	12	5–15	[28, 32, 33]
*γ*_H_	Infectious period in humans (days)	730	70–2920	[5, 28–31, 34, 39, 45]	60	21–365	[5, 28–31, 34, 39, 45]
*γ*_L_	Infectious period in animal (days)	50	50–182.5	[28, 32, 33]	50	50–182.5	[28, 32, 33]
*δ*_H_	Immune period in humans (days)	50	25–60	[5, 28–30]	50	25–60	[5, 28–30]
*δ*_L_	Immune period in animal (days)	50	25–60	[28, 32, 33]	50	25–60	[28, 32, 33]
*c*_V_	Tsetse migration rate (per day)	4x10^−4^	0–0.002	–	4x10^−4^	0–0.002	–

^a^Tsetse fecundity and mortality are density-dependent, and can be affected by intra-species competition [13]. Equations for tsetse birth and death rates can be found in S1 Text.

–Parameter values and ranges were assumed based on expert opinion.

In our model, susceptible human and/or animal hosts can be infected with trypanosomiasis following a bite by an infected tsetse fly. Infected hosts progress from latent to infectious stages of the disease at rates corresponding to the natural history of HAT. Hosts transition from the infectious compartment following treatment, hospitalization, or death. Tsetse flies follow similar stages of disease progression. Wild type tsetse that have recently emerged from pupae are susceptible to trypanosome infection and can only be infected during their first blood meal on an infected host [20]. Although research has demonstrated that nutritionally deprived or older flies can be infected with trypanosomes, non-teneral flies are expected to play a minor role in the epidemiology of trypanosome transmission [21]. Thus, we assume that flies that have not been infected during their first blood meal are no longer susceptible to trypanosome infection and proceed to the resistant compartment. The duration that tsetse are susceptible to trypanosome infection is also varied in sensitivity analysis, as described below. Infected flies progress from the latent to infectious stages at rates corresponding to the natural history of trypanosome infection in tsetse. Infected flies do not recover from infection and stay infectious for the remainder of their lives.

Tsetse population dynamics are modeled simultaneously with disease transmission. Wild type flies are colonized with wild type *Sodalis* but not with *Wolbachia*. Paratransgenic flies are colonized both with recombinant *Sodalis* and *Wolbachia*. The rates of reproduction of offspring that are colonized with *Sodalis* alone, *Wolbachia* alone, neither symbiont, or both symbionts are determined from a combination of tsetse fecundity, the extent of *Wolbachia*’s reproductive advantage, and the efficiency of the vertical transmission of the symbionts. Although symbiont density may vary within a tsetse host by age or mating status [22], our population-level model stratifies by presence or absence of symbionts within tsetse and does not account for within host variation.

Flies colonized with recombinant *Sodalis* are resistant to trypanosome transmission. Consequently, they do not contribute to disease transmission and are born into to the resistant vector compartment. Flies without recombinant *Sodalis* are born into the susceptible vector compartment and can be infected with trypanosomes at their first blood meal. Paratransgenesis is modeled by introducing into the resistant vector compartment a number of recombinant *Sodalis*-positive and *Wolbachia*-positive (*i*.*e*. paratransgenic) flies equal to 10% of the total wild type tsetse population size at the beginning of the model simulation.

Because paratransgenesis has not yet been developed for particular species of tsetse, we do not model a single species of tsetse. Model parameters that may differ among species, such as tsetse biting rates of humans and animals, are varied across ranges reported in the literature and calculated from both lab and field data for vectors of *T*.*b*. *gambiense* and *T*.*b*. *rhodesiense* (Table 1). In addition, model parameters for which precise values are not yet known, including paratransgenesis-specific parameters and migration rates, are varied across plausible ranges in the sensitivity analysis (Table 1).

Over the 25-year period of the model simulations, paratransgenic tsetse either become fixed in the population or went extinct. To reduce the reproductive number below one and sustain HAT elimination, we found that paratransgenic tsetse must become fixed at a prevalence greater than 84% for *T*.*b*. *gambiense* or 87% for *T*.*b*. *rhodesiense*. Fixation at prevalences below 84% or 87% will reduce the number of cases of *T*.*b*. *gambiense* or *T*.*b*. *rhodesiense*, respectively, but will not reduce the reproductive number below one and eliminate HAT. Thus, we used 84% and 87% paratransgenesis threshold as our criterion of HAT elimination of *T*.*b*. *gambiense* and *T*.*b*. *rhodesiense*, respectively.

### Sensitivity Analyses

We performed local sensitivity analyses on the paratransgenesis parameters to determine the biologically and epidemiologically feasible ranges (Table 1) over which HAT elimination could be achieved. When elimination was not feasible, we also considered whether a reduction in HAT incidence is achievable over the 25 year simulations. To determine which paratransgenesis parameters had the greatest influence on the percentage of human cases averted as a result of the release of paratransgenic tsetse, we performed global sensitivity analyses, including calculating the sensitivity index and partial rank correlation coefficient (PRCC). Sensitivity indices were calculated by fixing the value for the parameter of interest at a value from its distribution of likely values and the proportion of human cases averted was calculated while all other parameters were randomly sampled from their distributions of likely values [23]. Joint distributions of likely parameter values were drawn from biologically realistic ranges (Table 1), restricting possible parameter value combinations to those that produce an *R*_0_ for trypanosomiasis greater than one, indicating sustained HAT transmission in the absence of intervention, as we know to be the case. In the presence of paratransgenic intervention, we calculated the mean and variance of human cases averted across all possible values of the parameter of interest from 1,000 simulations. We calculated the sensitivity index as the ratio of the variance of the estimate to the total variance in all estimates of the proportion of human cases averted. Sensitivity index values closer to one indicated that a variable contributed more to the uncertainty in the proportion of cases averted estimates. The proportion of HAT cases averted over 25 years was then calculated by execution of the model for each of 10,000 Latin Hypercube samples from the parameter ranges in Table 1, with a further restriction of the joint distribution of likely parameter values to those combinations that yielded an *R*_0_ for trypanosomiasis greater than one. PRCCs were calculated to evaluate the monotonicity of the relationship between each parameter sampled and the consequent proportion of HAT cases averted.

## Results

We found that the fecundity and mortality penalties associated with *Wolbachia* or recombinant *Sodalis* colonization, the probability of vertical transmission, and tsetse migration rates are fundamental to determining whether HAT elimination is achieved via paratransgenesis. Parameter thresholds determined which of three possible outcomes occurred following the release of paratransgenic tsetse: complete HAT elimination, negligible impact on HAT burden, or partial reduction in HAT cases.

**Fig 2:**
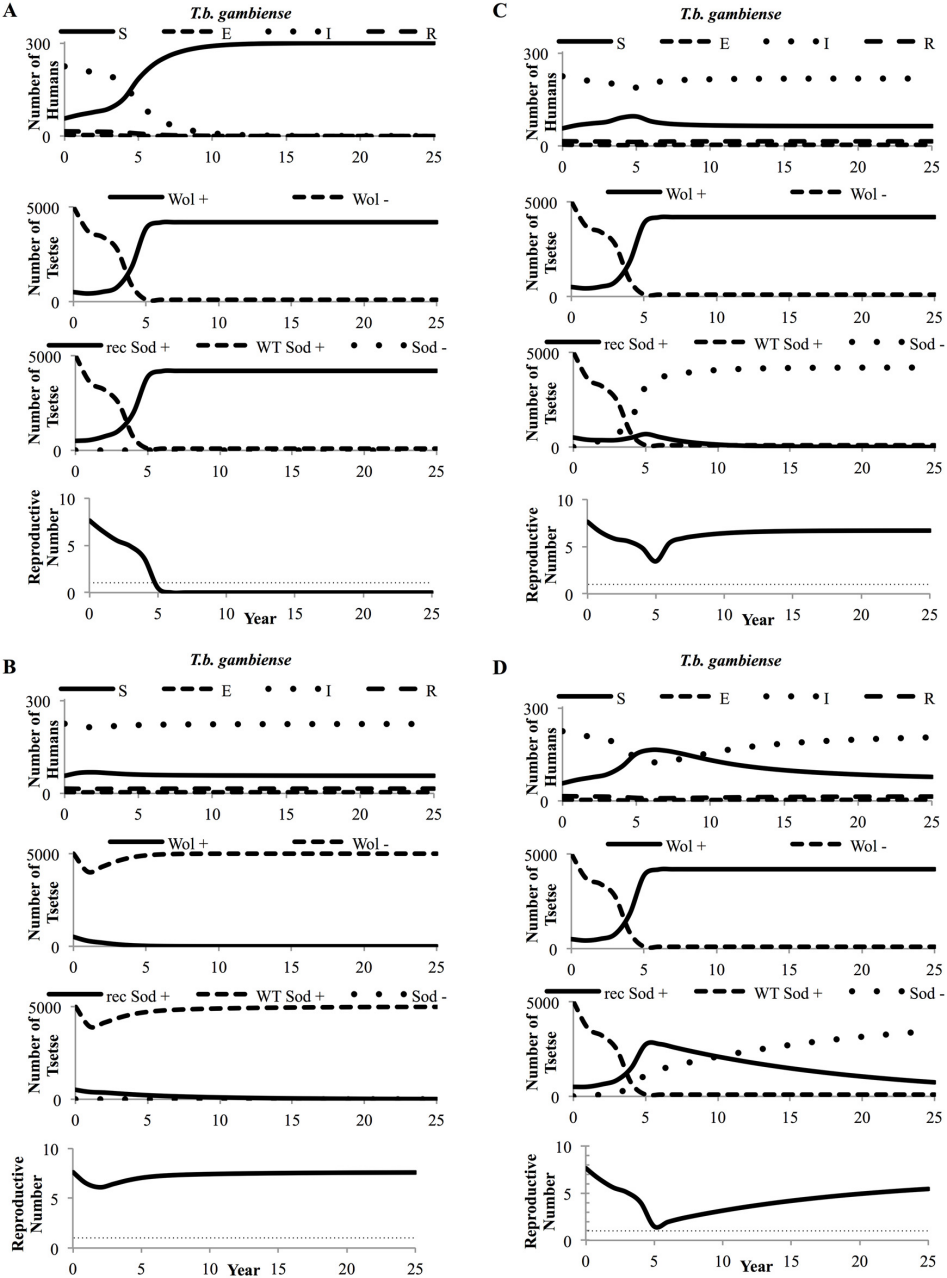
Tsetse population and *Trypanosoma brucei gambiense* dynamics 25 years following release of paratransgenic tsetse. **(A)** *T*.*b*. *gambiense* and perfect maternal transmission of recombinant *Sodalis* (0% vertical transmission failure), **(B)** *T*.*b*. *gambiense* and a 5% fecundity penalty for *Wolbachia* colonization, **(C)** *T*.*b*. *gambiense* and 5% probability of failure for maternal transmission of recombinant *Sodalis*, and **(D)** *T*.*b*. *gambiense* and 1% probability of failure for maternal transmission of recombinant *Sodalis*. S = Susceptible humans, E = Exposed humans, I = Infectious humans, R = Recovered humans, Wol + = *Wolbachia*-positive tsetse, Wol— = *Wolbachia*-negative tsetse, rec Sod + = recombinant *Sodalis*-positive tsetse, WT Sod + = wild type *Sodalis*-positive tsetse, and Sod— = *Sodalis*-negative tsetse.

**Fig 3:**
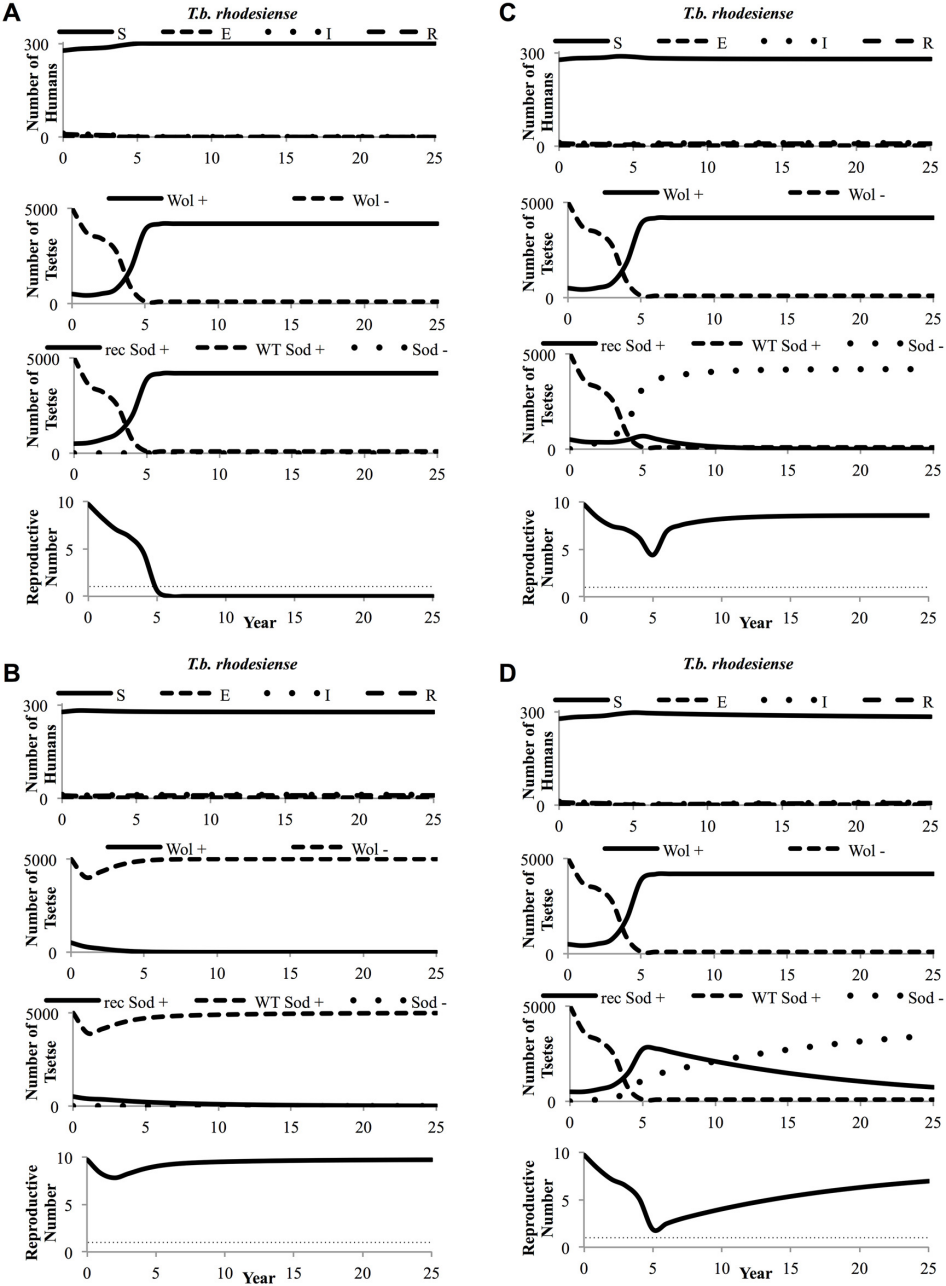
Tsetse population and *Trypanosoma brucei rhodesiense* dynamics 25 years following release of paratransgenic tsetse. **(A)** *T*.*b*. *rhodesiense* and perfect maternal transmission of recombinant *Sodalis* (0% vertical transmission failure), **(B)** *T*.*b*. *rhodesiense* and a 5% fecundity penalty for *Wolbachia* colonization, **(C)** *T*.*b*. *rhodesiense* and 5% probability of failure for maternal transmission of recombinant *Sodalis*, and **(D)** *T*.*b*. *rhodesiense* and 1% probability of failure for maternal transmission of recombinant *Sodalis*. S = Susceptible humans, E = Exposed humans, I = Infectious humans, R = Recovered humans, Wol + = *Wolbachia*-positive tsetse, Wol— = *Wolbachia*-negative tsetse, rec Sod + = recombinant *Sodalis*-positive tsetse, WT Sod + = wild type *Sodalis*-positive tsetse, and Sod— = *Sodalis*-negative tsetse.

Our results show that for paratransgenesis to eliminate HAT, *Wolbachia* must drive recombinant *Sodalis* into the tsetse population at a prevalence greater than 84% (for *T*.*b*. *gambiense* elimination) or 87% (for *T*.*b*. *rhodesiense* elimination), which will render enough flies resistant to trypanosome infection, thereby reducing the reproductive number for HAT below one (Figs 2A and 3A). A reproductive number less than one indicates that the spread of trypanosomiasis between flies, humans, and animals will eventually cease and the disease will be eliminated. When HAT is eliminated in this scenario, *T*.*b*. *gambiense* HAT 25 year cumulative incidence is reduced by 30% and *T*.*b*. *rhodesiense* HAT by 21% (Figs 2A and 3A).

**Fig 4:**
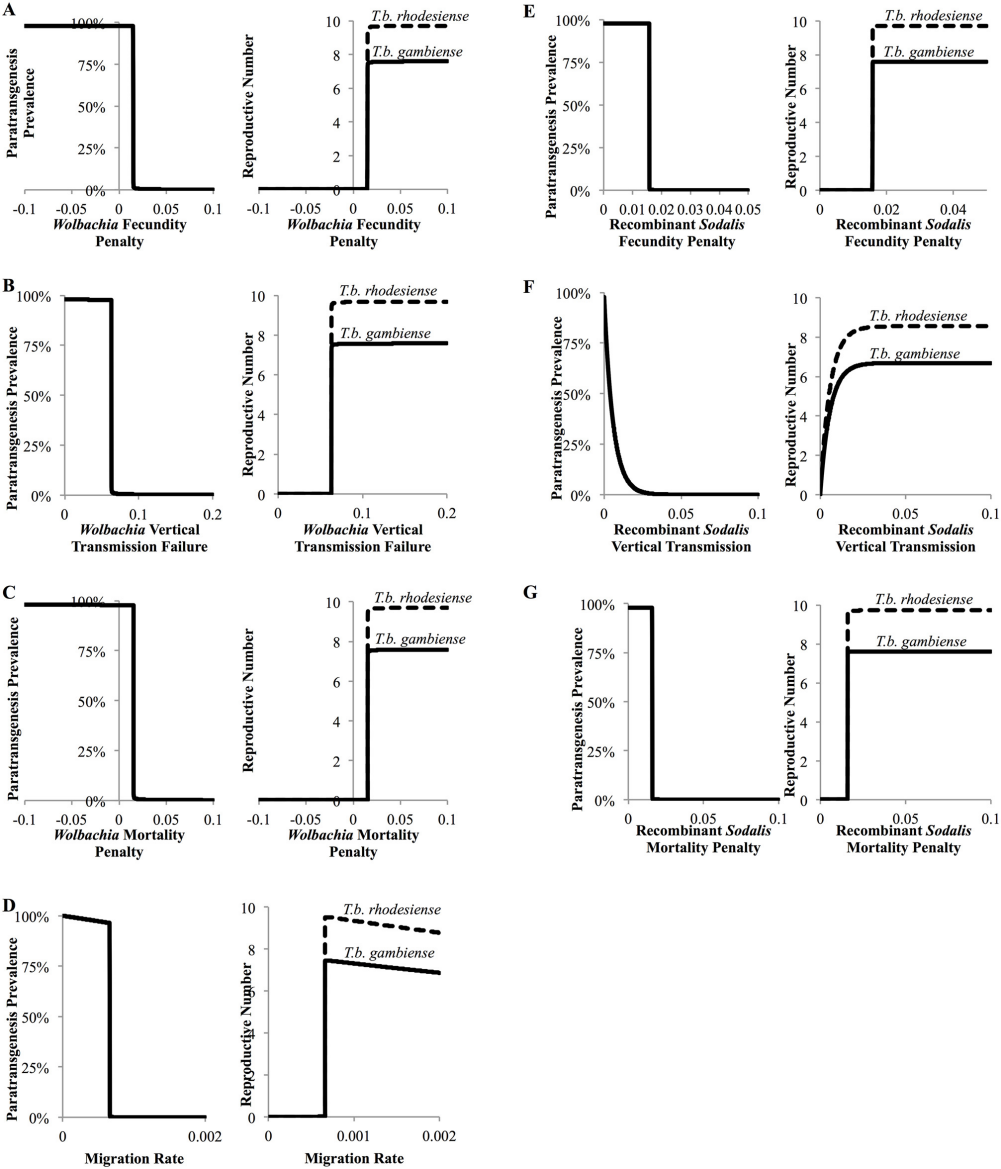
Local sensitivity analysis of paratransgenic parameters on the prevalence of paratransgenic tsetse and the reproductive number of *T*.*b*. *gambiense* and *T*.*b*. *rhodesiense*. **(A)** Fecundity penalty for *Wolbachia* infection, **(B)** Probability of maternal transmission failure for *Wolbachia*, **(C)** Increase in death rate (mortality penalty) due to *Wolbachia* infection, **(D)** Migration rate of tsetse, **(E)** Fecundity penalty for recombinant *Sodalis* colonization, **(F)** Probability of maternal transmission failure of recombinant *Sodalis*, and **(G)** Increase in death rate (mortality penalty) due to recombinant *Sodalis* colonization.

One-way sensitivity analysis identified several parameter thresholds that directly influenced whether paratransgenesis is likely to eliminate HAT. First, for *Wolbachia* to become fixed in the tsetse population and for the HAT reproductive number to be reduced below one, the *Wolbachia* fecundity penalty must be less than 1.5% (Fig 4A; *i*.*e*. 98.5% or more offspring of *Wolbachia* colonized tsetse successfully mature into adults), the probability of successful vertical transmission over 93.7% (Fig 4B; *i*.*e*. 6.3% or fewer of tsetse offspring are born without *Wolbachia*), the mortality penalty less than 1.5% (Fig 4C; *i*.*e*. *Wolbachia* colonized tsetse die at a rate no more than 1.5% greater than wild type tsetse), and the tsetse migration rate in and out of the population less than 65% above baseline migration (0.0007 or 3.5 tsetse in and out of the population of 5,000 per day; Fig 4D). Second, in order for recombinant *Sodalis* to be driven by *Wolbachia* into the tsetse population at a prevalence sufficient to reduce the HAT reproductive number to below one, recombinant *Sodalis* colonization must have a fecundity penalty no more than 1.6% (Fig 4E; *i*.*e*. 98.4% or more offspring of recombinant *Sodalis* colonized tsetse mature into adults), a probability of vertical transmission success greater than 99.9% (Fig 4F; *i*.*e*. fewer than 0.1% of offspring are born without recombinant *Sodalis*), and a mortality penalty of recombinant *Sodalis* colonization no greater than 1.6% (Fig 4G; *i*.*e*. recombinant *Sodalis* colonized tsetse die at a rate no higher than 1.6% that of wild type tsetse).

If the fitness penalty or migration rate parameter thresholds are exceeded, paratransgenesis will not eliminate or reduce HAT. Under these conditions, *Wolbachia* does not drive itself into the tsetse population and recombinant *Sodalis* also does not become fixed in the tsetse population (Figs 2B and 3B). Consequently, the number of tsetse colonized with recombinant *Sodalis* drops to zero quickly over a period of a few years, the HAT reproductive number remains above one, and the cumulative number of human or animal HAT infections is not reduced by more than a few percent. Another challenge can arise if recombinant *Sodalis* becomes unlinked from *Wolbachia* vertical transmission due to imperfect vertical transmission of recombinant *Sodalis* (Figs 2C and 3C). In this case, while *Wolbachia* becomes fixed in the tsetse population, trypanocidal recombinant *Sodalis* does not. Thus, the HAT reproductive number remains above one and cases are not reduced.

**Fig 5:**
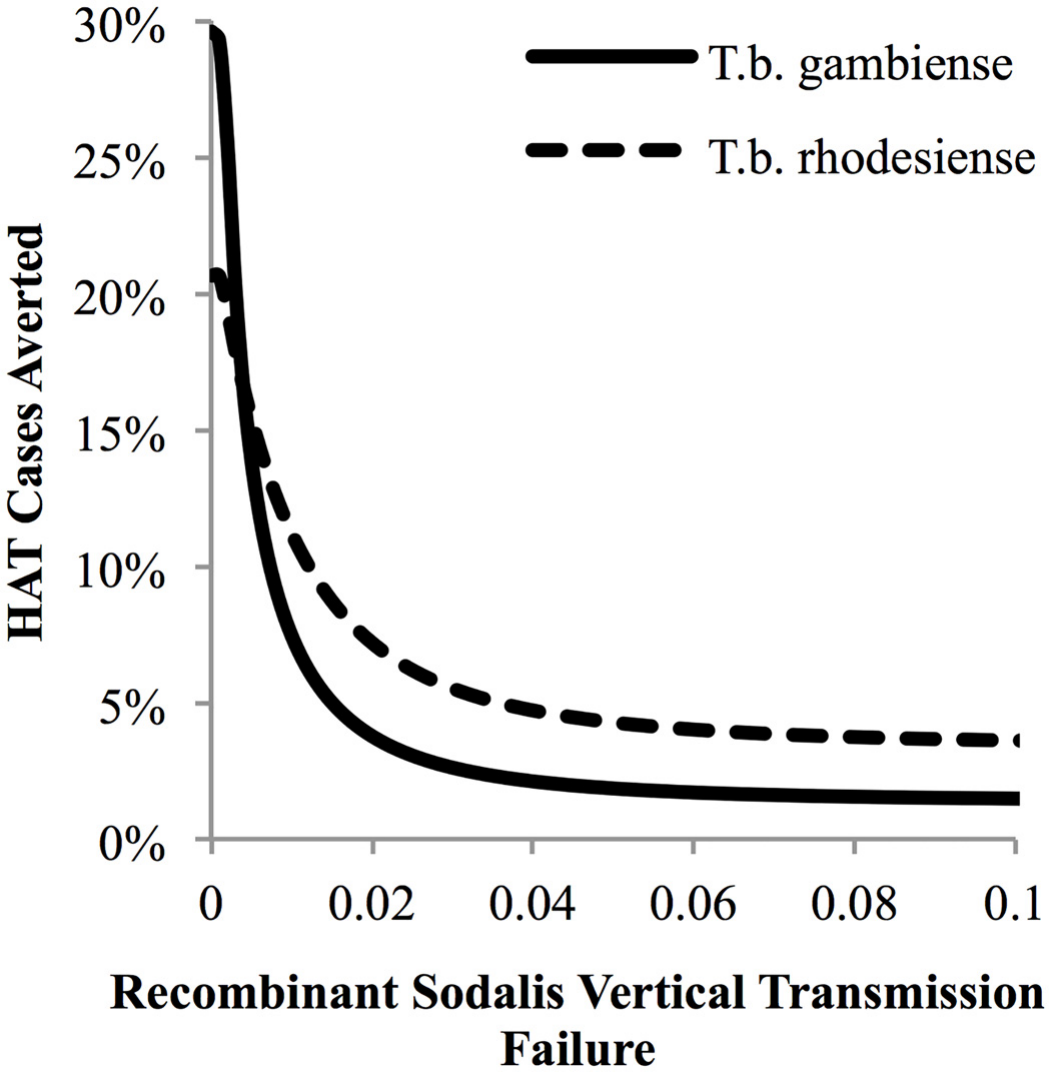
Local sensitivity analysis of probability that recombinant *Sodalis* will fail to be transmitted vertically on the percentage of *T*.*b*. *gambiense* and *T*.*b*. *rhodesiense* HAT cases averted by paratransgenesis over 25 years.

Local sensitivity analysis also identified parameter regions for which paratransgenesis could partially reduce HAT over 25-years without complete elimination. If the probability of recombinant *Sodalis* transmission failure from paratransgenic mother to offspring is greater than 0.1% but lower than 9.4% for *T*.*b*. *gambiense* or lower than 14.2% for *T*.*b*. *rhodesiense* (Fig 4F), recombinant *Sodalis* becomes partially, but not completely, unlinked from *Wolbachia* transmission (Figs 2D and 3D). In this parameter range *Wolbachia* still becomes fixed in the tsetse population at a prevalence close to 100%, but recombinant *Sodalis* becomes fixed at a lower prevalence between 84% (*T*.*b*. *gambiense*) or 87% (*T*.*b*. *rhodesiense*) and 0%. The HAT reproductive number is not reduced below one. When the probability of vertical transmission failure of recombinant *Sodalis* is less than 0.1%, approximately 30% of *T*.*b*. *gambiense* and 21% of *T*.*b*. *rhodesiense* HAT cases are averted (Fig 5). As the probability of vertical transmission failure is increased from 0% to 9.4%, the cumulative HAT cases averted drops to 1% for *T*.*b*. *gambiense*; when it is increased to 14.2%, the cumulative HAT cases averted drops to 3% for *T*.*b*. *rhodesiense*, indicating that HAT elimination is highly sensitive to the probability of recombinant *Sodalis* vertical transmission failure.

Global sensitivity analysis ranked the *Wolbachia* fecundity penalty as the most important model parameter for averting HAT cases. The analysis yielded a sensitivity index of 0.119 and PRCC of −0.716 (*P* < 0.001) for *T*.*b*. *gambiense* and a sensitivity index of 0.108 and PRCC of −0.733 (*P* < 0.001) for *T*.*b*. *rhodesiense* (Table 2). The remaining paratransgenesis parameters did not appreciably influence the percentage of HAT cases averted over 25 years, and had statistically significant PRCCs less than 0.25 and sensitivity indices equal to or less than 0.01 (Table 2). Therefore, ensuring that there is a low fecundity penalty for *Wolbachia* is paramount for HAT elimination to be achievable.

**Table 2:** Global sensitivity analysis of model parameters for both *Trypanosoma brucei* subspecies.

Parameter description	*T*.*b*. *gambiense*				*T*.*b*. *rhodesiense*			
	Sensitivity index	PRCC	P-value	Rank	Sensitivity index	PRCC	P-value	Rank
**Fecundity penalty for *Wolbachia* infection**	0.1190	–0.7155	<0.001	1	0.1078	–0.7328	<0.001	1
**Fraction of wild type tsetse that are inherently immune**	0.0135	–0.3714	<0.001	2	0.0120	–0.3680	<0.001	3
**Maternal transmission failure probability for *Wolbachia***	0.0123	–0.1293	<0.001	3	0.0107	–0.1203	<0.001	4
**Human population size**	0.0098	–0.3874	<0.001	4	0.0128	–0.3706	<0.001	2
**Fecundity density-dependence parameter**	0.0089	–0.2553	<0.001	5	0.0106	–0.2308	<0.001	5
**Migration rate (per day)**	0.0073	–0.2231	<0.001	6	0.0071	–0.2211	<0.001	6
**Transmission probability from tsetse to humans**	0.0061	0.2926	<0.001	7	0.0061	0.3303	<0.001	8
**Mortality increase due to *Wolbachia* infection**	0.0057	–0.2036	<0.001	8	0.0044	–0.2010	<0.001	9
**Maternal transmission failure probability for recombinant *Sodalis***	0.0049	–0.1857	<0.001	9	0.0043	–0.2112	<0.001	10
**Tsetse human biting rate (per day)**	0.0040	0.4100	<0.001	10	0.0061	0.4261	<0.001	7
**Mortality increase due to recombinant *Sodalis* infection**	0.0038	–0.1281	<0.001	11	0.0032	–0.1279	<0.001	12
**Livestock population size**	0.0038	0.0651	0.001	12	0.0042	0.1130	<0.001	11
**Susceptibility period in tsetse (days)**	0.0033	–0.2663	<0.001	13	0.0030	–0.2711	<0.001	15
**Tsetse fecundity (per day)**	0.0030	–0.0239	0.233	14	0.0029	–0.0022	0.916	16
**Mortality density-dependence parameter**	0.0027	–0.1953	<0.002	15	0.0031	–0.2348	<0.001	13
**Infectious period humans (days)**	0.0025	0.0152	0.448	16	0.0011	–0.0472	0.021	24
**Transmission probability from livestock to tsetse**	0.0025	0.2154	<0.001	17	0.0030	0.2250	<0.001	14
**Fraction of *Sodalis* negative tsetse that are inherently immune**	0.0022	0.1581	<0.001	18	0.0021	0.1442	<0.001	17
**Fecundity penalty for recombinant *Sodalis* infection**	0.0019	–0.0683	0.001	19	0.0017	–0.0475	0.021	20
**Fecundity penalty for no *Sodalis* infection**	0.0018	0.1143	<0.001	20	0.0017	0.109	<0.001	19
**Tsetse mortality**	0.0017	–0.1298	<0.001	21	0.0019	–0.1789	<0.001	18
**Cytoplasmic incompatibility hatch failure probability**	0.0014	0.0723	<0.001	22	0.0013	0.0475	0.021	21
**Tsetse livestock biting rate (per day)**	0.0011	0.178	0.374	23	0.0012	0.0092	0.654	22
**Incubation time tsetse (days)**	0.0011	0.0168	0.401	24	0.0011	0.0282	0.170	26
**Immune period humans (days)**	0.0011	0.0028	0.891	25	0.0010	–0.0270	0.188	31
**Incubation time humans (days)**	0.0011	–0.0001	0.996	26	0.0010	–0.0371	0.071	29
**Mortality increase due to no *Sodalis* infection**	0.0010	0.0171	0.395	27	0.0011	–0.0075	0.716	27
**Transmission probability from tsetse to livestock**	0.0010	–0.0186	0.353	28	0.0011	–0.0191	0.353	23
**Immune period in livestock (days)**	0.0010	0.0411	0.040	29	0.0011	0.0308	0.134	25
**Incubation time livestock (days)**	0.0010	0.0063	0.754	30	0.0010	0.0197	0.337	28
**Infectious period livestock (days)**	0.0010	–0.0814	<0.001	31	0.0010	–0.0336	0.102	32
**Transmission probability from humans to tsetse**	0.0010	0.0038	0.851	32	0.0010	0.0008	0.970	30
**Maternal transmission failure probability for wild type *Sodalis***	0.0009	–0.0211	0.292	33	0.0010	–0.0149	0.467	33

## Discussion

To investigate how the dynamics of *Wolbachia* and recombinant *Sodalis* impact HAT elimination via paratransgenesis, we modeled the dynamics of *T*.*b*. *gambiense* and *T*.*b rhodesiense* infection in humans, animals, and tsetse, while also incorporating the population dynamics of tsetse symbiotic bacteria *Wolbachia* and *Sodalis* into our model. We found that HAT elimination was highly dependent on thresholds for fitness penalties associated with paratransgenesis, inefficiencies in symbiont transmission, and tsetse migration. When these parameters were below their threshold values, *Wolbachia* and recombinant *Sodalis* became fixed in the tsetse population at a prevalence close to 100% over a period of 25 years, rendering the majority of the tsetse population resistant to trypanosome infection and thereby eliminating *T*.*b*. *gambiense* and *T*.*b*. *rhodesiense* cases. If either *Wolbachia* or recombinant *Sodalis* did not become fixed in the population at a prevalence high enough to reduce the reproductive number below one, as was the case when the thresholds for paratransgenesis parameters are exceeded, HAT was not eliminated. Furthermore, the thresholds for elimination did not vary by trypanosome species, despite differences between *T*.*b*. *gambiense* and *T*.*b*. *rhodesiense* regarding prevalence and duration of infection within humans and animals, as well as regarding vector preference for feeding on humans versus animals.

We found that fecundity and mortality penalties associated with *Wolbachia* colonization most influenced the successful fixation of paratransgenic tsetse and reduction in HAT cases. When these penalties are too high or vertical transmission from mother to offspring is not efficient, *Wolbachia* fails to serve as a driver of recombinant *Sodalis* into the tsetse population. The migration of wild type tsetse into the modeled population and paratransgenic tsetse out of the population also significantly impacted HAT elimination. When migration rates are too high, paratransgenic tsetse density is diluted by wild type tsetse, and HAT is not eliminated. The likelihood of HAT elimination is also sensitive to the fecundity and mortality penalties of recombinant *Sodalis*, as well as to vertical transmission probability. When penalties are too high or vertical transmission is inefficient, recombinant *Sodalis* becomes unlinked from *Wolbachia* vertical transmission, and paratransgenic tsetse fails to eliminate HAT. In addition, we determined parameter regions for which HAT elimination is not likely, but for which a reduction in incidence could be sustained. Specifically, when vertical transmission is between 99.9% and 90.6% (for *T*.*b*. *gambiense*) or 85.8% (for *T*.*b*. *rhodesiense*), recombinant *Sodalis* could become fixed in the tsetse population at a prevalence between 0% and 84% (*T*.*b*. *gambiense*) or 87% (*T*.*b*. *rhodesiense*) over the course of 25 years, thereby partially reducing cumulative *T*.*b*. *gambiense* HAT incidence between 30% and 1% and *T*.*b*. *rhodesiense* HAT incidence between 26% and 3%, respectively.

Although paratransgenesis has the potential to successfully eliminate HAT, the high sensitivity of *Wolbachia* and recombinant *Sodalis* prevalence to symbiont fitness penalties and tsetse migration rates indicates that these properties are fundamental to the effectiveness of paratransgenic approaches developed to curtail HAT. Given that HAT elimination is most sensitive to *Wolbachia* parameters, it will be essential to select a *Wolbachia* subspecies that has minimal, if any, fitness penalties in tsetse. *Wolbachia* infections are heterogeneous in natural populations, and prevalence has been found to vary from 0% to 100% [12, 24, 25]. In the laboratory, *Wolbachia* infections have been established in multiple tsetse species with a prevalence of 100% [12, 24, 25]. The successful elimination of HAT via paratransgenesis will not only necessitate the selection of *Wolbachia* species that have minimal fitness penalties in tsetse, but also tsetse species that are both well-suited to the ecology of targeted location and able to host a *Wolbachia* infection.

Moreover, it will also be critical to ensure that the recombinant *Sodalis* strain is transmitted from mother to offspring with a nearly 100% probability, and that it imposes only minor fecundity or mortality penalties. There is evidence that unmodified *Sodalis* alone may affect the fecundity or mortality of some tsetse species [14, 26, 27]. Of particular importance is the engineering of recombinant *Sodalis* to produce a molecule that effectively targets trypanosomes without simultaneously reducing the fitness of *Sodalis*. These characteristics of the selected *Wolbachia* strain and engineered recombinant *Sodalis* will need to be rigorously tested to ensure that HAT is successfully eliminated. If it becomes apparent that high vertical transmission probabilities for recombinant *Sodalis* cannot be achieved, the potential for partial reduction in HAT incidence will need to be investigated to determine if the benefit is nonetheless worth the cost of the intervention. Furthermore, if recombinant *Sodalis* transmission inefficiencies cause paratransgenic tsetse to be established at a prevalence in the population lower than the 84% or 87% required for elimination of *T*.*b*. *gambiense* or *T*.*b*. *rhodesiense*, respectively, it may be possible to time subsequent releases of paratransgenic flies—each using different subspecies of *Wolbachia*—to continue to drive recombinant *Sodalis* into the tsetse population. Finally, because migration rates may vary by location and tsetse species, paratransgenesis should be targeted to areas with low rates of tsetse migration. Low migration, combined with trapping of wild type flies prior to the intervention, maximizes the relative density of paratransgenic flies, ensuring the greatest number of HAT cases are averted while paratransgenic flies are prevalent, even if HAT elimination is not achieved.

## Supporting Information

S1 Text This document contains an appendix of additional model description and equations. (DOCX)
