# Supplementary material for: Determinants of Human African Trypanosomiasis Elimination via Paratransgenesis
Source: PLoS Negl Trop Dis. 2016 Mar 8;10(3):e0004465. doi: 10.1371/journal.pntd.0004465 (PMC4783105; doi:10.1371/journal.pntd.0004465)
Supplement: S1 Text — (DOCX) [file pntd.0004465.s001.docx]

**Determinants of Human African Trypanosomiasis Elimination via Paratransgenesis**

**Supporting Information Text S1**

Jennifer A. Gilbert^1,2*^, Jan Medlock^3^, Jeffrey P. Townsend^4,5,6^, Serap Aksoy^2^, Martial Ndeffo Mbah^1,2¶^, and Alison P. Galvani^1,2¶^

^1^Center for Infectious Disease Modeling and Analysis, Yale School of Public Health, New Haven, Connecticut, United States of America

^2^Epidemiology of Microbial Diseases, Yale School of Public Health, New Haven, Connecticut, United States of America

^3^Department of Biomedical Sciences, Oregon State University, Corvallis, Oregon, United States of America

^4^Department of Biostatistics, Yale University, New Haven, Connecticut, United States of America

^5^Department of Ecology and Evolutionary Biology, Yale University, New Haven, Connecticut, United States of America

^6^Program in Computational Biology and Bioinformatics, Yale University, New Haven, Connecticut, United States of America

^*^ Corresponding author

E-mail: [jennifer.gilbert@aya.yale.edu](mailto:jennifer.gilbert@aya.yale.edu) (JAG)

^¶^These authors contributed equally to this work.

***Wolbachia* and *Sodalis* Tsetse Population Dynamics and Equations**

Here we describe the population dynamics of tsetse, including vertical transmission of *Wolbachia* and *Sodalis.* Trypanosome transmission between tsetse, humans, and animals is described separately in another section below.

Let *V*_pr_ be the number of tsetse positive for both *Wolbachia* and recombinant *Sodalis*, *V*_pn_ be the number positive for *Wolbachia* and negative for *Sodalis*, *V*_nw_ be the number negative for *Wolbachia* and positive for wild type *Sodalis*, *V*_nr_ be the number negative for *Wolbachia* and positive for recombinant *Sodalis*, and *V*_nn_ be the number negative for both *Wolbachia* and *Sodalis.* Then let *V*_po_ = *V*_pr_ + *V*_pn_ be the number of *Wolbachia*-positive tsetse, *V*_no_ = *V*_nr_ + *V*_nw_ + *V*_nn_ be the number of *Wolbachia*-negative tsetse, *V*_or_ = *V*_pr_ + *V*_nr_ be the number of recombinant *Sodalis*-positive tsetse, *V*_ow_ = *V*_nw_ be the number of wild type *Sodalis-*positive tsetse, *V*_on_ = *V*_pn_ + *V*_nn_ be the number of *Sodalis-*negative tsetse, and *V* = *V*_pr_ + *V*_pn_ + *V*_nr_ + *V*_nw_ + *V*_nn_ be the total number of tsetse.

Let $r_{V}\left( V \right)$ be the fecundity of *Wolbachia*-negative, wild type *Sodalis*-positive tsetse ($V_{nw}$) as a non-increasing function of tsetse population size. Let ${1-r}_{W}$ be the relative fecundity of *Wolbachia*-positive tsetse, ${1-r}_{Sr}$ be the relative fecundity of recombinant *Sodalis*-positive tsetse, ${1-r}_{Sn}$ be the relative fecundity of *Sodalis*-negative tsetse, and the three factors be multiplicative and independent of tsetse population size. Let $\mu_{V}\left( V \right)$ be the death rate of *Wolbachia*-negative, wild type *Sodalis*-positive tsetse as a non-decreasing function of tsetse population size. Let ${1+\mu}_{Sw}$ be the relative death rate of *Wolbachia*-positive tsetse, ${1+\mu}_{Sr}$ be the relative death rate of recombinant *Sodalis*-positive tsetse, ${1+\mu}_{Sn}$1+muSn be the relative death rate of *Sodalis*-negative tsetse, and the three factors be multiplicative and independent of tsetse population size. Let $\varphi_{W}$ be the proportion of eggs of *Wolbachia*-positive mothers that are *Wolbachia*-negative, and $\varphi_{Sr}$ be the proportion of eggs of recombinant *Sodalis*-positive mothers that are *Sodalis*-negative. Finally, let $h_{W}$ be the proportion of *Wolbachia*-negative eggs fertilized by *Wolbachia*-positive males that do not mature and develop.

The following system of Ordinary Differential Equations describes the population dynamics:

$\frac{dV_{pr}}{dt}=\left[ r_{V}\left( V \right)\left( 1-r_{W} \right)\left( 1-r_{Sr} \right)\left( 1-\phi_{W} \right)\left( 1-\phi_{Sr} \right) \right.$ (1) $\left. -\mu_{V}\left( V \right)\left( 1+\mu_{W} \right)\left( 1+\mu_{Sr} \right)+c_{V} \right]V_{pr}$,

$$\frac{dV_{pn}}{dt}=r_{V}\left( V \right)\left( 1-r_{W} \right)\left( 1-r_{Sr} \right)\left( 1-\phi_{W} \right)\phi_{Sr}V_{pr}$$

$+\left[ r_{V}\left( V \right)\left( 1-r_{W} \right)\left( 1-r_{Sn} \right)\left( 1-\phi_{W} \right)-\mu_{V}\left( V \right)\left( 1+\mu_{W} \right)\left( 1+\mu_{Sn} \right)-c_{V} \right]V_{pn}$, (2)

$$\frac{dV_{nr}}{dt}=r_{V}\left( V \right)\left( 1-r_{W} \right)\left( 1-r_{Sr} \right)\phi_{W}\left( 1-\phi_{Sr} \right)\left( 1-h_{W}\frac{V_{po}}{V} \right)V_{pr}$$

$+\left[ r_{V}\left( V \right)\left( 1-r_{Sr} \right)\left( 1-\phi_{Sr} \right)\left( 1-h_{W}\frac{V_{po}}{V} \right)-\mu_{V}\left( V \right)\left( 1+\mu_{Sr} \right)-c_{V} \right]V_{nr}$, (3)

$\frac{dV_{nw}}{dt}=c_{V}\left( V \right)+\left[ r_{V}\left( V \right)\left( 1-\phi_{Sw} \right)\left( 1-h_{W}\frac{V_{po}}{V} \right)-\mu_{V}\left( V \right)-c_{V} \right]V_{nw}$, (4)

$$\frac{dV_{nn}}{dt}=r_{V}\left( V \right)\left( 1-r_{W} \right)\left( 1-r_{Sr} \right)\phi_{W}\phi_{Sr}\left( 1-h_{W}\frac{V_{po}}{V} \right)V_{pr}$$

$+ r_{V}\left( V \right)\left( 1-r_{W} \right)\left( 1-r_{Sn} \right)\phi_{W}\left( 1-h_{W}\frac{V_{po}}{V} \right)V_{pn}$

$$+ r_{V}\left( V \right)\left( 1-r_{W} \right)\left( 1-r_{Sr} \right)\phi_{Sr}\left( 1-h_{W}\frac{V_{po}}{V} \right)V_{nr}+r_{V}\left( V \right)\phi_{Sw}\left( 1-h_{W}\frac{V_{po}}{V} \right)V_{nw}$$

$+\left[ r_{V}\left( V \right)\left( 1-r_{Sn} \right)\left( 1-h_{W}\frac{V_{po}}{V} \right)-\mu_{V}\left( V \right)\left( 1+\mu_{Sn} \right)-c_{V} \right]V_{nn}$, (5)

where the density-dependent birth rate function is

$r_{V}\left( V \right)=r_{V0}\left( 1-r_{V1}V \right)$. (6)

The parameter $r_{V0}$ is the birth rate in the absence of intra-species competition, while $r_{V1}$ measures the effect of intra-species competition on birth rate.

The density-dependent mortality rate function is

$\mu_{V}\left( V \right)=\mu_{V0}\left( 1-\mu_{V1}V \right)$. (7)

The parameter $\mu_{V0}$ is the death rate in the absence of intra-species competition, while $\mu_{V1}$ measures the effect of intra-species completion on death rate.

If there are no fecundity and death rate penalties for colonization with either *Wolbachia* or *Sodalis* (or if the whole tsetse population is negative for both bacteria), the equilibrium population size is then $V^{*}=\frac{r_{V0}-\mu_{V0}}{r_{V0}r_{V1}+\mu_{V0}\mu_{V1}}$. With fecundity or death rate penalties, this provides an upper bound on the tsetse population size.

**Trypanosome Infection Model and Equations**

We assume that the presence of recombinant *Sodalis* renders a tsetse fly completely immune to trypanosome infection. Therefore, in the infection model we only consider wild type *Sodalis*-positive and *Sodalis*-negative tsetse, *V*_ow_ and *V*_on_. Let *V*_S_ be the number of susceptible tsetse, *V*_E_ be the number of exposed tsetse, *V*_I_ be the number of infectious tsetse, and *V*_R_ be the number of resistant tsetse. Note that *V*_S_ + *V*_E_ + *V*_I_ + *V*_R_ = *V*_ow_ + *V*_on_. Let *H*_S_, *H*_E_, *H*_I_, and *H*_R_ be the number of susceptible, exposed, infectious, and recovered humans. Let *L*_S_, *L*_E_, *L*_I_, and *L*_R_ be the number of susceptible, exposed, infectious, and recovered animals.

Tsetse with wild type *Sodalis* (*V*_ow_) emerge from pupae according to equations 1 through 7 described above, with a fraction $\psi_{Vw}$ of newly emerged *V*_ow_ inherently resistant to trypanosome infection and the remaining ${1-\psi}_{Vw}$ susceptible. Likewise, tsetse without *Sodalis* (*V*_on_) emerge with a fraction $\psi_{Vn}$ inherently resistant and the remaining ${1-\psi}_{Vn}$ susceptible. We assume that susceptible tsetse become infected with trypanosomes with probabilities $\lambda_{VH}$ and $\lambda_{VL}$ during their blood meals taken on humans and animals, respectively, within a certain window of length ($\frac{1}{\sigma_{V}}$) following emergence. Blood meals are taken at rates $\alpha_{H}$ and $\alpha_{L}$ on humans and animals, respectively, and the conservation of law of tsetse bites applies, where the number of bites taken by tsetse balances the total number of bites on hosts, applies. Tsetse trypanosome infections incubate for a period of $\frac{1}{\tau_{V}}$, after which point they become infectious. Tsetse can migrate in or out of any state at rate $c_{V}$. Finally, tsetse die at rate $\hat{\mu}_{V}$.

(8)

$\frac{dV_{E}}{dt}=\left(a_{H}\lambda_{VH}+a_{L}\lambda_{VL}\right)V_{S}-\left(\tau_{V}+\hat{\mu}_{V}+c_{V}\right)V_{E}$ (9)

$\frac{dV_{I}}{dt}=\tau_{V}V_{E}-\left(\hat{\mu}_{V}+c_{V}\right)V_{I}$ (10)

$\frac{dV_{R}}{dt}=B_{VR}+\left[a_{H}\left(1-\lambda_{VH}\right)+a_{L}\left(1-\lambda_{VL}\right)+\sigma_{V}\right]V_{S}-\left(\hat{\mu}_{V}+c_{V}\right)V_{R}$ (11)

$\frac{dH_{S}}{dt}=\delta_{H}H_{R}-a_{H}\lambda_{H}\frac{H_{S}}{H}$ (12)

$\frac{dH_{E}}{dt}=a_{H}\lambda_{H}\frac{H_{S}}{H}-\tau_{H}H_{E}$ (13)

$\frac{dH_{I}}{dt}=\tau_{H}H_{E}-\gamma_{H}H_{I}$ (14)

$\frac{dH_{R}}{dt}=\gamma_{H}H_{I}-\delta_{H}H_{R}$ (15)

$\frac{dL_{S}}{dt}=\delta_{L}L_{R}-a_{L}\lambda_{L}\frac{L_{S}}{L}$ (16)

$\frac{dL_{E}}{dt}=a_{L}\lambda_{L}\frac{L_{S}}{L}-\tau_{L}L_{E}$ (17)

$\frac{dL_{I}}{dt}=\tau_{L}L_{E}-\gamma_{L}L_{I}$ (18)

$\frac{dL_{R}}{dt}=\gamma_{L}L_{I}-\delta_{L}L_{R}$ (19)

where

$\lambda_{VH}=\beta_{VH}\frac{H_{I}}{H}$, (20)

$\lambda_{VL}=\beta_{VL}\frac{L_{I}}{L}$,

$\lambda_{H}=\beta_{H}V_{I}$,

$$\lambda_{L}=\beta_{L}V_{I}.$$

From Equations 1 through 7, the tsetse birth terms are

$B_{V_{ow}}^{*}=r_{V}\left( V^{*} \right)\left( 1-\phi_{Sw} \right)\left( 1-h_{W}\frac{V_{po}^{*}}{V^{*}} \right)V_{nw}^{*}$, (21)

$$B_{V_{on}}^{*}=r_{V}\left( V^{*} \right)\left( 1-r_{W} \right)\left( 1-r_{Sr} \right)\phi_{Sr}\left( 1-\phi_{W}h_{W}\frac{V_{po}^{*}}{V^{*}} \right)V_{pr}^{*}$$

$+ \left( 1-r_{W} \right)\left( 1-\phi_{W}h_{W}\frac{V_{po}^{*}}{V^{*}} \right)V_{pn}^{*}+\left( 1-r_{Sr} \right)\phi_{Sr}\left( 1-h_{W}\frac{V_{po}^{*}}{V^{*}} \right)V_{nr}^{*}$

$+ \phi_{Sw}\left( 1-h_{W}\frac{V_{po}^{*}}{V^{*}} \right)V_{nw}^{*}+\left( 1-r_{Sn} \right)\left( 1-h_{W}\frac{V_{po}^{*}}{V^{*}} \right)V_{nn}^{*}$

$+\frac{c_{V}\left( V^{*} \right)\hat{\mu}_{V}^{*}}{a_{H}+a_{L}+\sigma_{V}+\hat{\mu}_{V}^{*}}$, (22)

$B_{VS}^{*}=\left( 1-\psi_{V_{w}} \right)B_{V_{ow}}^{*}+\left( 1-\psi_{V_{n}} \right)B_{V_{on}}^{*}$, (23)

$B_{VR}^{*}=\psi_{V_{w}}B_{V_{ow}}^{*}+\psi_{V_{n}}B_{V_{on}}^{*}$, (24)

From Equations 1 through 7, the tsetse death rate is

$\hat{\mu}_{V}^{*}=\mu_{V}\left( V^{*} \right)\left( 1+\frac{\mu_{W}V_{pn}^{*}+\mu_{Sn}V_{nn}^{*}}{V_{ow}^{*}+V_{on}^{*}} \right)$. (25)

**Reproduction Number**

For our model equations, the basic reproduction number for trypanosome infection is calculated by the next-generation matrix [1], counting one generation as tsetse-to-tsetse transmission. This gives

$R_{0}=\left[ \frac{a_{H}^{2}\beta_{H}\beta_{VH}}{\gamma_{H}H}+\frac{a_{L}^{2}\beta_{L}\beta_{VL}}{\gamma_{L}L} \right]\frac{\tau_{V}}{\left( \hat{\mu}_{V}^{*}+c_{V} \right)\left( \tau_{V}+\hat{\mu}_{V}^{*}+c_{V} \right)}\frac{B_{VS}^{*}}{a_{H}+a_{L}+\sigma_{V}+\hat{\mu}_{V}^{*}+c_{V}}$. (26)

**References**

1. van den Driessche P, Watmough J. Reproduction numbers and sub-threshold endemic equilibria for compartmental models of disease transmission. Mathematical Biosciences. 2002;180:29-48.

2. Rogers DJ. A general model for the African trypanosomiases. Parasitology. 1988;97:193-212.

3. Alam U, Medlock J, Brelsfoard C, Pais R, Lohs C, Balmand S, et al. Wolbachia symbiont infections induce strong cytoplasmic incompatibility in the tsetse fly Glossina morsitans. PLoS pathogens. 2011;7(12):e1002415. doi: 10.1371/journal.ppat.1002415. PubMed PMID: 22174680; PubMed Central PMCID: PMC3234226.

4. Artzrouni M, Gouteux JP. Control strategies for sleeping sickness in Central Africa: a model-based approach. Tropical medicine & international health : TM & IH. 1996;1(6):753-64. PubMed PMID: 8980586.

5. Artzrouni M, Gouteux JP. A compartmental model of sleeping sickness in central Africa. J Biol Sys. 1996;4(4):459-77.

6. Chalvet-Monfray K, Artzrouni M, Gouteux JP, Auger P, Sabatier P. A two-patch model of Gambian sleeping sickness: application to vector control strategies in a village and plantations. Acta biotheoretica. 1998;46(3):207-22. PubMed PMID: 10220868.

7. Habtemariam T, Ruppanner R, Riemann HP, Theis JH. An Epidemiologic Systems Analysis Model for African Trypanosomiasis. Prev Vet Med. 1982/1983;1:125-36.

8. Habtemariam T, Ruppanner R, Riemann HP, Theis JH. Epidemic and Endemic Characteristics of Trypanosomiasis in Cattle: A Simulation Model. Prev Vet Med. 1982/1983;1:137-45.

9. Muller G, Grebaut P, Gouteux JP. An agent-based model of sleeping sickness: simulation trials of a forest focus in southern Cameroon. Comptes rendus biologies. 2004;327(1):1-11. PubMed PMID: 15015750.

10. McDermott JJ, Coleman PG. Comparing apples and oranges--model-based assessment of different tsetse-transmitted trypanosomosis control strategies. International journal for parasitology. 2001;31(5-6):603-9. PubMed PMID: 11334949.

11. Milligan PJ, Baker RD. A model of tsetse-transmitted animal trypanosomiasis. Parasitology. 1988;96 ( Pt 1):211-39. PubMed PMID: 3362578.

12. Trail JC, Murray M, Sones K, Jibbo JM, Durkin J, Light D. Boran cattle maintained by chemoprophylaxis under trypanosomiasis risk. J Agric Sci. 1985;105(1):147-66.

13. Baker RD. Modelling trypanosomiasis prevalence and periodic epidemics and epizootics. IMA journal of mathematics applied in medicine and biology. 1992;9(4):269-87. PubMed PMID: 1302761.

14. Clausen PH, Adeyemi I, Bauer B, Breloeer M, Salchow F, Staak C. Host preferences of tsetse (Diptera: Glossinidae) based on bloodmeal identifications. Medical and veterinary entomology. 1998;12(2):169-80. PubMed PMID: 9622371.

15. Negash M, Girma M, Seyoum E. Epizootiological importance of Glossina morsitans submorsitans (Diptera: Glossinidae) (Newstead) in the Ghobe River Valley, Southwest Ethiopia. Acta Trop. 2007;102(2):100-5.

16. Fevre EM, Picozzi K, Jannin J, Welburn SC, Maudlin I. Human African Trypanosomiasis: Epidemiology and Control. Adv Parisitol. 2006;61(167-221).

17. Checchi F, Filipe JA, Haydon DT, Chandramohan D, Chappuis F. Estimates of the duration of the early and late stage of gambiense sleeping sickness. BMC infectious diseases. 2008;8:16. doi: 10.1186/1471-2334-8-16. PubMed PMID: 18261232; PubMed Central PMCID: PMC2259357.
